# DNA methylation alterations in acute lymphoblastic leukemia survivors with late neurocognitive deficits

**DOI:** 10.1038/s41375-025-02779-0

**Published:** 2025-10-17

**Authors:** Sarah J. Goodman, Darci T. Butcher, Sharon L. Guger, Eric Diehl, Jack Brzezinski, Brenda Spiegler, Brian J. Nieman, Prajkta Kallurkar, Aubrée Boulet Craig, Maja Krajinovic, Julie Laniel, Caroline Laverdière, Daniel Sinnett, Sarah Lippé, Andrei Turinsky, Mary Shago, Lisa J. Strug, Shinya Ito, Johann K. Hitzler, Russell Schachar, Rosanna Weksberg

**Affiliations:** 1 Genetics and Genome Biology, SickKids Research Institute, Toronto, ON Canada; 2 Department of Pathology and Molecular Medicine, McMaster University, Hamilton, VIC Canada; 3 Department of Psychology, The Hospital for Sick Children, Toronto, ON Canada; 4 Department of Paediatric Laboratory Medicine, The Hospital for Sick Children, Toronto, ON Canada; 5 Department of Pediatrics Division of Haematology and Oncology, The Hospital for Sick Children, Toronto, ON Canada; 6 Mouse Imaging Centre, The Hospital for Sick Children, Toronto, ON Canada; 7 Department of Medical Biophysics, University of Toronto, Toronto, ON Canada; 8 Centre for Computational Medicine, The Hospital for Sick Children, Toronto, ON Canada; 9 Sainte-Justine University Health Center, Research Center, Montréal, QC Canada; 10 Department of Psychology, Université de Montréal, Montréal, QC Canada; 11 Department of Pediatrics, Université de Montréal, Montréal, QC Canada; 12 The Centre for Applied Genomics, SickKids Research Institute, Toronto, ON Canada; 13 Departments of Statistical Sciences and Computer Science, University of Toronto, Toronto, ON Canada; 14 Developmental and Stem Cell Biology, The Hospital for Sick Children Research Institute, Toronto, ON Canada; 15 Division of Clinical Pharmacology and Toxicology, The Hospital for Sick Children, Toronto, ON Canada; 16 Department of Psychiatry, The Hospital for Sick Children, Toronto, Canada ON; 17 Neurosciences and Mental Health, SickKids Research Institute, Toronto, ON Canada; 18 Division of Clinical and Metabolic Genetics, the Hospital for Sick Children, Toronto, ON Canada; 19 Department of Molecular Genetics, University of Toronto, Toronto, ON Canada

**Keywords:** Cancer genomics, Risk factors

## To the Editor:

While 5-year survival rates for acute lymphoblastic leukemia (ALL) approach 90%, [1, 2], many ALL survivors develop adverse late effects months to years after completion of therapy, which severely impact their quality of life [3–7]. Neurocognitive deficits are common, often impacting attention and executive function and manifesting as learning and behavioral problems, which can lead to poor educational achievement, unemployment, and lower quality of life [7, 8]. Despite the removal of cranial radiotherapy (CRT) from standard CNS prophylaxis due to its known link to long-term neurocognitive deficits [9], estimates of neurocognitive deficits remain at 25–40% in ALL survivors treated with chemotherapy only [10–12]. This raises questions of the mechanistic link between chemotherapy and late effects and highlights the need to understand and predict individual risks for neurocognitive impairments following ALL therapy to provide more personalized treatments and interventions [13, 14].

DNA methylation (DNAm) has recently been proposed as a possible molecular underpinning of late effects based on a growing body of evidence of chemotherapy-related DNAm changes. In a study of over 2000 adult survivors of childhood cancer, an epigenome-wide association analysis identified over 1000 CpG sites associated with exposure to specific chemotherapy agents or radiotherapy, with a subset of these associated with cardiometabolic late effects [15]. Evidence of robust DNAm differences between cancer survivors, irrespective of late effects, and individuals with no history of cancer or exposure to chemotherapeutic agents is still needed as foundational evidence on which to build the study of epigenetic biomarkers and mechanisms of late effects. Using multiple independent cohorts of ALL survivors, survivors of other childhood cancers, and healthy individuals with no history of cancer, we identified a genome-wide DNAm signature comprised of altered DNAm levels specific to ALL survivors.

N-PhenoGENICS (NPG) is a study of ALL survivors based at SickKids Hospital, Toronto, Canada(16 in Supplementary methods and materials); participants ranged from 8–20 years of age, were diagnosed with ALL between the ages of 0.7–13 years and included individuals treated with only chemotherapy. The NPG cohort of ALL survivors (*n* = 140) was separated into discovery (*n* = 91) and validation sets (*n* = 49), such that training samples were balanced with healthy control samples for age, sex, and technical batch (Supplementary Table S1).

**Fig. 1:**
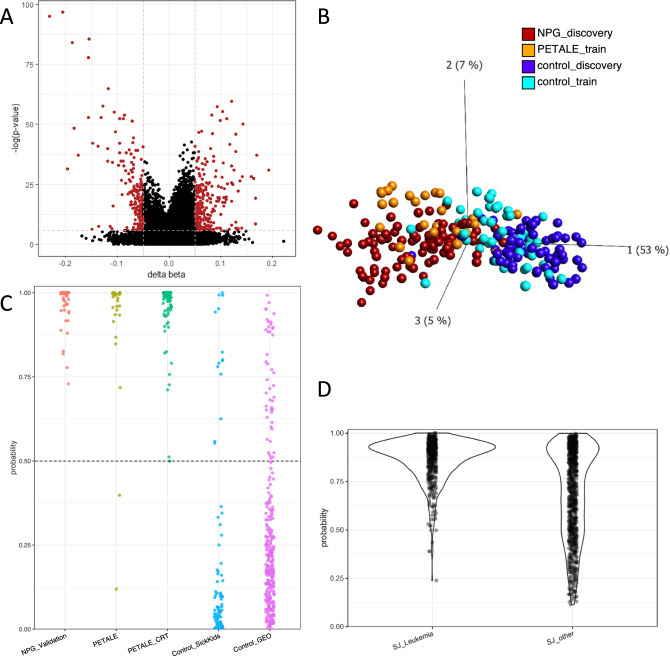
differentially methylated CpGs between ALL survivors in the NPG discovery group and healthy controls and predictive efficacy of differentially methylated sites. **A** 452 CpGs (red) that met genome-wide significance (*q*-value < 0.05; db > 5%) in testing for differential methylation between ALL survivors (NPG discovery) and controls. **B** PCA at 33 CpGs, derived from filtering the 452 differentially methylated CpGs, cluster controls from ALL survivors in NPG discovery (*n* = 91) and controls (*n* = 70), and indepedent samples (PETALE all survivors without CRT, *n* = 25 and additional control samples, *n* = 50). PC1 accounts for 53% of variability across samples at these 33 CpGs. All samples plotted were used as training samples of RF model. **C** Probability scores from RF model demonstrate 98% sensitivity and 85% specificity in 110 additional ALL survivors (PETALE, PETALE CRT) and 394 controls (controls Weksberg and control GEO). All NPG validations (*n* = 49) were classified correctly. The threshold was set to 50% as indicated by the dashed line. **D** Applying the classification model to St. Jude's LIFE cohort (GSE169156), with the cohort split into two groups. “SJ leukemia” (*n* = 510) were individuals presumed to be treated for leukemia or lymphoma and “SJ other” (*n* = 1023) were individuals treated for all other cancers reported in this cohort. RF probability scores in SJ leukemia (99% positive) and SJ other (77%) were significantly different.

Running an epigenome-wide analysis identified a signature of 452 differentially methylated CpGs between the NPG discovery cases and controls (*q*-value < 0.05, Δβ > 5%; Fig. 1; Supplementary Fig. S1; Table S2); 38% of sites (173 CpGs) exhibited hypomethylation in the ALL survivors, as compared to controls. Of the 452 CpG signature sites differentially methylated between ALL survivors and healthy controls, 154 had been previously reported by Song et al. as correlating with exposure to one or more chemotherapy agents in the St. Jude LIFE study population (Song et al. reported 652 unique CpGs total) [15]. These findings support the idea that lasting molecular, including epigenetic, and physiological changes, likely result not from a single chemotherapy agent, but rather a combination of multiple agents.

We trained a random forest (RF) classification model to assess the sensitivity and specificity of DNAm to predict past treatment for ALL; training samples included NPG and control discovery samples described above, in addition to 25 ALL survivors from PETALE with no CRT exposure, and 50 additional healthy controls (Supplementary Table S1). The RF classification model was trained using DNAm patterns at 33 CpGs, filtered from the full 452 CpGs, that demonstrated robust DNAm differences between survivors and healthy controls and could appropriately cluster samples (Fig. 1B, see Supplementary methods and materials). For each sample, the classifier produced a probability of a DNAm profile originating from an ALL survivor, with scores above 0.5 were deemed “ALL-survivor-like” and scores below that threshold were “control-like”. The model demonstrated 98% sensitivity when applied to the remaining 110 ALL survivors from PETALE (note: all 49 NPG validation samples were correctly classified), and 85% specificity when applied to 394 healthy controls (Fig. 1C, Supplementary Fig. S2, Supplementary Table S1). Age at the time of sample collection was correlated with the length of time since treatment ended (in years) but had no association with probability scores, nor did age at diagnosis, or CRT (Supplementary Fig. S3), suggesting that the DNAm associated with treatment did not weaken over time.

PBMC-derived DNAm data from the St. Jude LIFE cohort, restricted to age 40 years or younger, were separated into two groups for which we inferred primary diagnosis based on the chemotherapy treatment. Group 1 (*n* = 510) was predicted “leukemia/lymphoma” group based on treatment with vinca alkaloids, asparaginase, antimetabolites, and corticosteroids; 200 of these individuals received CRT. Group 2 (*n* = 1023) included all remaining samples, which consisted of primary diagnoses of sarcoma, CNS tumors, embryonal tumors and “other” (Supplementary Table S1) [15]. Using an RF model trained on an expanded CpG set (AUC > 80 filter reduced to AUC > 70), 99% of the leukemia/lymphoma samples were correctly classified as ALL-survivor-like (504 of 510), while significantly fewer (77%) of the “other” samples were classified as ALL-survivor-like (789 of 1023; Wilcoxin *p* < 0.05; Fig. 1D). This finding indicated that a similar but demonstrably different DNAm profile was present in PBMCs of these two groups of cancer survivors, years to decades after treatment.

**Fig. 2:**
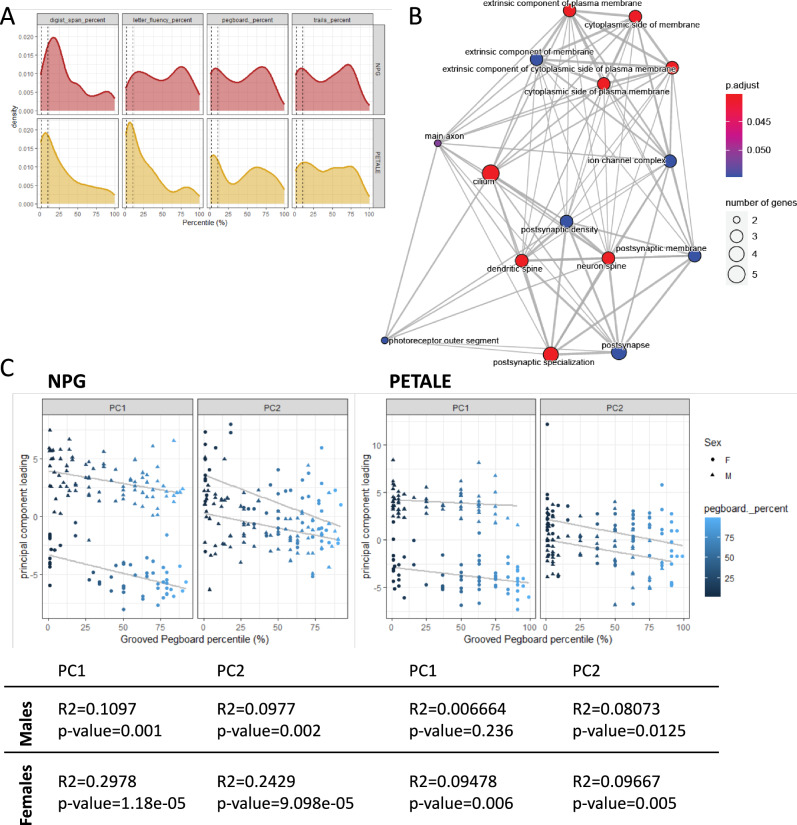
Associations between neurocognitive deficits and DNA methylation in two cohorts of ALL survivors, NPG and PETALE. **A** Percentile of DIVERGT test scores with dotted lines indicating the 2^nd^ and 10^th^ percentile, which were used to categorize neurocognitive deficits or low performance (40% of the PETALE cohort and 30% of the NPG cohort). The difference in scores between NPG (red) and PETALE (yellow) was not significant at any test. Population scores for all tests are normally distributed about the 50^th^ percentile. **B** Significant GO enrichment terms (13 terms) from genes mapping to the 43 CpGs significantly associated with one or more DIVERGT test scores in meta-analysis. **C** PC1 and PC2 of 43 CpGs in NPG (left) and PETALE (right) showing associations with grooved pegboard scores (as percentiles) and sex. Statistical measures correspond to the PC and cohort in the plot above.

To measure neurocognitive impairments, specifically in executive functioning, participants of PETALE and NPG were assessed on the DIVERGT test battery (Fig. 2A). In PETALE, DIVERGT was previously reported to accurately predict General Ability Index, mathematics, and verbal memory in adult ALL survivors (17 in Supplementary methods and materials). Within each cohort, we found low to moderate pairwise correlations between the four subtests, Digit Span (WAIS/WISC/IV), Verbal Fluency (D-KEFS), Grooved Pegboard, and Trail Making Test (D-KEFS); the strongest correlation in both cohorts was between Digit Span and Trail Making Test (NPG r = 0.42, PETALE r = 0.49; Supplementary Fig. S4). “Low performance”, defined by scoring below the 2^nd^ percentile on one or more tests, or scoring below the 10^th^ percentile on two or more tests, was found in 40% (*n* = 54) of individuals in the PETALE cohort and 30% (*n* = 42) of NPG, commensurate with current estimates of ALL-treatment-related neurocognitive deficits (18–20 in Supplementary methods and materials).

To test for DNAm correlates of executive functioning, we applied a linear regression to each of the four DIVERGT tests against DNAm levels at each CpG, separately in each cohort, and applied a meta-analysis to identify CpGs independently associated with the same test score in both PETALE and NPG. Digit Span scores were associated with 2 CpGs, Verbal Fluency (D-KEFS) scores were associated with 1 CpG, and Grooved Pegboard was significantly associated with 43 CpGs (*q*-values for all CpGs < 0.05; Supplementary Table S3). As expected, based on the interrelatedness of the test scores, there was overlap in these sets and the 43 CpGs associated with Grooved Pegboard were inclusive of the CpGs associated with other tests. These CpGs mapped to genes significantly enriched for 13 GO terms (Fig. 2B), which organized into two clusters relating to neuronal cell features such as “dendritic spine” and cell adhesion; these ontologies were composed of genes including, *SHANK3*, *DNM1*, and *CADPS2*, each of which displayed loss of DNA methylation in the gene body in correlation with lower DIVERGT scores. Dendritic spines specifically are thought of as the structural basis of learning and memory, and therefore play a critical role in executive functioning.

The Grooved Pegboard scores were significantly lower in males (Student’s t *p*-value < 0.05; Supplementary Fig. S5) in the PETALE cohort and trended in the same direction in the NPG cohort. A PCA of the 43 Grooved Pegboard sites did in fact show separation between males and females on PC1, as well as a relationship between PC1 scores and Pegboard scores (Fig. 2C). PC2 also exhibited an association with less delineation between sexes. Within each sex, PC1 and PC2 loadings were significantly associated with test scores in the NPG cohort (Fig. 2D). Together, these findings illustrated a modest but reproducible relationship between Grooved Pegboard scores and DNAm levels in the blood of ALL survivors.

While the *p*-values were significant, the DNAm levels account for only a small proportion of the variation in Grooved Pegboard scores. As a biomarker, using DNAm to predict individuals at the highest risk of neurocognitive deficits would still be highly valuable even if the model was subject to some noise. Moreover, the biological mechanism of late effects we aimed to identify does not encompass all variation in cognition but only the contribution of past chemotherapy to this variation. Therefore, we expected that a large proportion of Grooved Pegboard scores, i.e., the population-based distribution, would not be accounted for in our model and would not be relevant to predicting depressed scores related to late effects.

In conclusion, we propose DNAm as a potential biomarker that could predict patients most likely to experience neurocognitive deficits following ALL treatment. We also suggest a link between cytotoxic chemotherapy and neurocognitive outcomes, at least in part, via differential methylation at genes regulating dendritic morphology and synaptogenesis. Given the prevalence of ALL, we expect it will be feasible in future studies to build on the findings presented here. These could target the collection of large cohorts to support studies stratified by sex, and also address risk protocol, primary diagnosis, and timing of the establishment of DNAm marks associated with neurocognitive outcomes or other common late effects. Ultimately, this work may inform the exploration of rational therapy modifications to mitigate late effects in ALL survivors.

## Supplementary information

Supplementary methods and materials

Supplementary Table 2

Supplementary Table 3

## Data Availability

The datasets discussed in this publication have been deposited in NCBI’s Gene Expression Omnibus and are accessible through GEO Series accession numbers GSE308314 and GSE308517 for NPG and PETALE, respectively.
