# Supplementary material for: DNA methylation alterations in acute lymphoblastic leukemia survivors with late neurocognitive deficits
Source: Leukemia. 2025 Oct 17;39(12):3042–6. doi: 10.1038/s41375-025-02779-0 (PMC12634443; doi:10.1038/s41375-025-02779-0)
Supplement: Supplementary file 1 — Supplementary methods and materials [file 41375_2025_2779_MOESM1_ESM.docx]

**SUPPLEMENTAL METHODS**

**Study Cohorts**

N-PhenoGENICS (NPG) is a study of ALL survivors based at SickKids Hospital, Toronto, Canada. Participants underwent deep phenotyping of neurocognitive and behavioural late effects in ALL survivors (See Methods Table S1. for inclusion and exclusion criteria). Pilot studies were run to identify risk factors of late effects and to elucidate molecular pathophysiology for neurocognitive deficits (1); a second round of recruitment expanded the study cohort and participants recruited from both phases were included here. One individual who was treated for Burkitt lymphoma was excluded from training and discovery analyses (See “DNAm analysis in samples derived from blood” for a description of these analyses). Children were treated using COG or CCG protocols: AALL0331, AALL0232, AALL0932, POG9904/9905 were most common (2-5).

**Methods Table S1 NPG inclusion and exclusion criteria**

| **NPG Inclusion** | **NPG Exclusion** |
| --- | --- |
| - Treated for ALL at SickKids between 1990-2010* - 8yr 0mths – 20yr 11mths at time of study visit - At least 2yrs from last ALL treatment - Blood draw for DNAm analysis taken at time of cognitive testing - Fluent in English - Received standard- or high-risk therapy | - History of stem cell transplant - History of cranial irradiation - Down syndrome - Language or cognitive barriers or inability to complete phenotype testing |

The second cohort of ALL survivors was independently collected at Sainte Justine University Health Center, Montreal, Canada, as part of the PETALE study (6). ALL survivors who participated were required to be at least 5 years post diagnosis and with no history of refractory or relapsed disease, or stem cell transplant. All participants were treated on DFCI-ALL 87-01 to 2005-01 protocols and 85 of the 135 PETALE participants included in this study were treated with CRT. The study was approved by SJUHC Institutional Review Board. Informed consent/assent was obtained from all NPG and PETALE participants and consent from their parents/guardians, when indicated (1, 6, 7).

With regards to differences in treatment protocols received by participants in NPG and PETALE, all patients received systemic and intrathecal chemotherapy including steroids, methotrexate, vincristine, anthracyclines, mercaptopurine, and asparaginase. Patients in the PETALE cohort were treated on DFCI consortium trials and in general, received higher cumulative doses of asparaginase. Patients in the NPG cohort were treated on COG trials and these therapies commonly included varying doses of alkylators and cytarabine. For all participants, irrespective of cohort, the cumulative dose intensity was determined on disease prognostic factors and both cohorts included both individuals treated with low and low/standard risk protocols and individuals treated with high risk protocols. The most notable difference in therapy was the addition of CRT for some PETALE participants. Given the demonstrated neurocognitive effects of CRT, individuals in PETALE who received this treatment were analyzed as a separate test group. Time of sampling was also measured differently between the cohorts, i.e. at least 5 years since diagnosis for PETALE vs. at least 2 years from end of chemotherapy for NPG, but overlapped (Methods Fig. S1). However, NPG participants were required to be 20 years or younger, while PETALE including individuals up to 40 years of age; given this difference and known associations between chronological age and DNA methylation, age was both including as a covariate in the linear regression, and assessed post hoc for collinearities with classifier predictions.


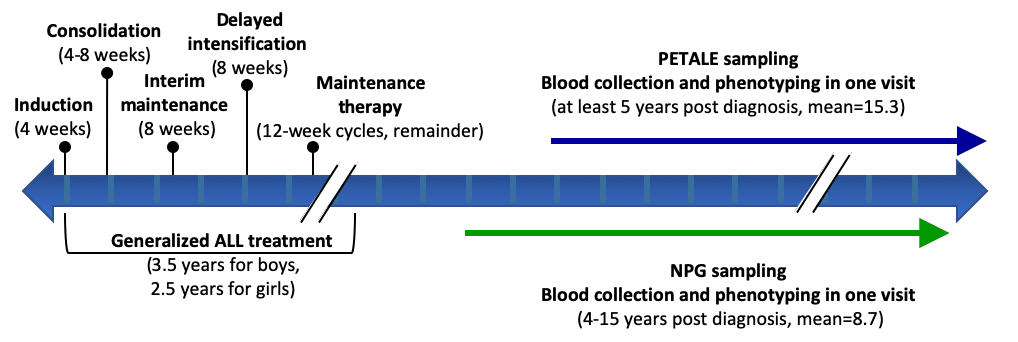


**Methods Fig. S1 Timeline sample collection and phenotyping relative to chemotherapy for NPG and PETALE cohorts.**

The third independent cohort of childhood cancer survivors was St. Jude LIFE study (GEO; GSE169156) included n=2052 adult survivors of childhood cancer who were treated at St. Jude Children’s Research Hospital (8, 9).

Multiple control cohorts were utilized, including1) samples collected through the SickKids Hospital, Toronto, Canada and 2) “GEO controls”, accessed through GEO (GSE147221) and which were only used in the testing phase (10). The latter were previously identified as healthy controls in an epigenome-wide analysis of individuals with schizophrenia and controls (10). We contacted the corresponding authors to obtain the raw data in the form of IDAT files. Only samples identified as controls were used.

**DNA methylation assay**

Converted DNA was then assayed on the Illumina Infinium MethylationEPIC array V1 (EPIC array) at The Center for Applied Genomics (TCAG), SickKids Hospital Research Institute, Toronto, Ontario, Canada in accordance with the manufacturer’s protocols. Samples were run in four three technical batches: batches 1 and 2 each included NPG blood samples and healthy controls; and batch 3 included all PETALE samples. Raw data processing from IDAT files and normalization were performed for all samples analyzed, including NPG, PETALE and publicly available data, using identical methods. This process was completed independently for each cohort: 1. All NPG blood samples and controls (run in two technical batches); 2. PETALE samples; 3. St. Jude LIFE; 4. GEO control samples; 5. previously assayed SickKids control samples. Cohorts were run separately through the processing pipeline since the Illumina normalization technique uses a “reference” array or sample; to that end, this reference sample is different for each cohort to prevent the normalization process from decreasing cohort differences that would impact generalization of the results to other populations.

**DNAm analysis in samples derived from blood**

As part of the preprocessing pipeline, proportions of monocytes, neutrophils, CD4^+^T, CD8^+^T, natural killer cells and B cells were estimated from the DNAm data separately in each cohort. This estimate was calculated using the function “estimateCellCounts2” from the “FlowSorted.Blood.EPIC” package and is based on the Houseman algorithm, a well-established and validated algorithm of blood DNAm proportions widely used in DNAm research (11). For this calculation, the unnormalized data were used and the EPIC reference platform was called. Plots were generated in R using package “ggplot2” (12) or Qlucore Omics Explorer (QOE, [www.qlucore.com](http://www.qlucore.com/)).

The first step of data analysis was separating the NPG blood samples and matched controls into a discovery group and validation group. The former would be used for signature discovery and model training, and the latter would be classified using said model to optimize filtering and other parameters prior to classifying all test samples. To derive the signature, a subset of 91 of 140 NPG samples was selected using the R package “MatchIt” to match to 70 controls; these were used in discovery (13). The “Matchit” function used the default method of nearest neighbor matching to subset the NPG cases to those best matched to controls for our input covariates of sex, age, and technical batch. In both the NPG discovery and validation group, proportions of CD4+T cells, estimated using DNAm levels, were significantly lower than in the controls (q-value<0.05; Methods Fig. S2). No other cell type differed in proportion between groups when p-values were corrected for multiple testing.

**Methods Fig. S2 Blood cell estimates in controls (n=70) and ALL survivors split into two groups: NPG discovery (n=91) and NPG validation (n=49).** ALL survivors demonstrated significantly lower CD4T cell proportions (NPG discovery vs control adjusted p=0.00026; NPG validation vs control adjusted p=0.00033).

At each CpG site, a two-group comparison of NPG discovery cases vs. controls was performed using limma regression, accounting for sex, age, technical batch and estimated blood cell proportion (CD8T, CD4T, natural killer cells, monocytes, and neutrophils) as covariates (14). CpG sites found to be differentially methylated between cases and controls were reported if they met both a statistical significance [false discovery rate (FDR)-corrected P-value < 0.01] and a minimum effect size (absolute Δb >10%); Δb represents the difference in average DNAm (b) between groups. Using the Illumina IDs, we compared the differentially methylation CpGs identified to those previously reported as associated with chemotherapy agents in survivors of childhood cancer (8).

Samples were then selected for machine learning model training. For model development, including training and sample selection, we implemented “best practices” for DNAm biomarkers development as outlined in Yousefi *et al.* (2022) (15). The samples used to identify differentially methylated CpGs were only those run in technical batches balanced for cases and controls, however, this was not necessary for model training. To that end, in addition to the NPG and controls samples used in discovery, a subset of PETALE and SickKids controls were selected for model training as described in the Methods.

Prior to running machine learning, filtering of the signature CpGs included removing CpGs significantly associated with any covariates in the linear model and those previously reported to be associated with smoking (16, 17), and those not meeting area-under-the-ROC-curve (AUC) threshold. The latter was generated separately on each CpG, treating the methylation level as a univariate predictor of the binary outcome, and CpGs with AUC<0.8 were removed prior to training the model to classify all PETALE and NPG samples (33 CpGs remained). This AUC threshold was lowered to >0.7 for training the model to classifying the St. Jude LIFE dataset as only 12 of the 33 filtered CpGs were available in this dataset following data processing and quality control. The relaxed AUC threshold resulted in 54 CpGs (12 CpGs used in previous RF model and 42 additional CpGs). A random forest (RF) model was trained on AUC>0.8 filtered CpGs using the NPG and control discovery samples and the additional PETALE and control training samples described above. Classifier development was performed using the caret package in R, which implements an internal cross-validation framework (18). Specifically, we applied 10-fold cross-validation during training to optimize hyperparameters and to evaluate model performance. For the St. Jude dataset we re-trained a RF model on the same training samples as described above. We employed the RF algorithm because it has been widely used in developing clinically-relevant classifiers from DNA methylation data, including in the Heidelberg Brain Tumour Classifier (19).

**Neurocognitive Assessments**

DIVERGT is a short battery of tests that has been shown to be predictive of both global 
and academic impairment in survivors of childhood cancer (20). The DIVERGT battery is a neuropsychological assessment comprised of performance-based measures of attention, working memory, speed of processing, fine motor control and set shifting, and takes approximately 20 minutes to complete. The tests included are: digit span from the age-appropriate Wechsler test, D-KEFS Verbal Fluency, grooved pegboard, and the D-KEFS Trail-Making Test (conditions 1-4).

**Meta-analysis**

Prior to DNA methylation analysis with the DIVERGT test scores, individuals with outlying scores were identified separately in each test and each cohort and were removed. Methods Table S2 contains the sample sizes after outlier filtering used in each linear model of test scores predicting DNAm values. Thresholds for identifying outliers were calculated using interquartile range criterion:

I=[q_0.25_−1.5⋅IQR;q_0.75_+1.5⋅IQR]

**Methods Table S2 Sample sizes used in linear regression analysis against DNA methylation**

|  | NPG | PETALE |
| --- | --- | --- |
| Trail Making test | 140 | 135 |
| Letter Fluency | 140 | 134 |
| Digit Span | 135 | 135 |
| Grooved Pegboard | 136 | 134 |

We then followed the approach published in Hannon *et al.* (2021) to run the meta-analysis and the publicly available script (<https://github.com/ejh243/SCZEWAS>) (10). First, we ran four linear models in each cohort, PETALE and NPG, using each of the four DIVERGT test z-scores as the main effect while covarying for sex, age, neutrophil proportion and technical batch in the NPG model and sex, age, neutrophil proportion, and CRT presence/absence in the PETALE model. From these we extracted standard error (SE) and Δβ for each CpG and ran four meta-analyses, again one for each DIVERGT test, using the “metagen” function in the “meta” package (21). P-values were corrected for multiple testing using FDR, correcting for the number of CpGs tested in each meta-analysis. Significant CpGs, met an FDR-corrected p-value<0.05, identified in the random effects model.

**GO enrichment analysis**

GO analysis Gene ontology (GO) enrichment analysis was performed on the 43 CpG sites significantly associated with one or more DIVERGT test. The R package ClusterProfiler was used to first translate the gene names available in the “UCSC_RefGene_name” column of the Illumina manifest to Entrez IDs (22). The function “enrichGO” was then run to identify enriched GO terms. Terms in any sub-ontologies, i.e. cellular component, molecular function, or biological process, that had an FDR corrected p-value<0.1 were reported.

**SUPPLEMENTAL METHODS REFERENCES**

1. Van Der Plas E, Erdman L, Nieman BJ, Weksberg R, Butcher DT, O'Connor D L, et al. Characterizing neurocognitive late effects in childhood leukemia survivors using a combination of neuropsychological and cognitive neuroscience measures. Child Neuropsychol. 2018;24(8):999-1014.

2. Maloney KW, Devidas M, Wang C, Mattano LA, Friedmann AM, Buckley P, et al. Outcome in Children With Standard-Risk B-Cell Acute Lymphoblastic Leukemia: Results of Children's Oncology Group Trial AALL0331. J Clin Oncol. 2020;38(6):602-12.

3. Larsen EC, Devidas M, Chen S, Salzer WL, Raetz EA, Loh ML, et al. Dexamethasone and High-Dose Methotrexate Improve Outcome for Children and Young Adults With High-Risk B-Acute Lymphoblastic Leukemia: A Report From Children's Oncology Group Study AALL0232. J Clin Oncol. 2016;34(20):2380-8.

4. Angiolillo AL, Schore RJ, Kairalla JA, Devidas M, Rabin KR, Zweidler-McKay P, et al. Excellent Outcomes With Reduced Frequency of Vincristine and Dexamethasone Pulses in Standard-Risk B-Lymphoblastic Leukemia: Results From Children's Oncology Group AALL0932. J Clin Oncol. 2021;39(13):1437-47.

5. Winick N, Martin PL, Devidas M, Shuster J, Borowitz MJ, Paul Bowman W, et al. Randomized assessment of delayed intensification and two methods for parenteral methotrexate delivery in childhood B-ALL: Children's Oncology Group Studies P9904 and P9905. Leukemia. 2020;34(4):1006-16.

6. Marcoux S, Drouin S, Laverdiere C, Alos N, Andelfinger GU, Bertout L, et al. The PETALE study: Late adverse effects and biomarkers in childhood acute lymphoblastic leukemia survivors. Pediatr Blood Cancer. 2017;64(6).

7. Boulet-Craig A, Robaey P, Laniel J, Bertout L, Drouin S, Krajinovic M, et al. DIVERGT screening procedure predicts general cognitive functioning in adult long-term survivors of pediatric acute lymphoblastic leukemia: A PETALE study. Pediatr Blood Cancer. 2018;65(9):e27259.

8. Song N, Hsu CW, Pan H, Zheng Y, Hou L, Sim JA, et al. Persistent variations of blood DNA methylation associated with treatment exposures and risk for cardiometabolic outcomes in long-term survivors of childhood cancer in the St. Jude Lifetime Cohort. Genome Med. 2021;13(1):53.

9. Hudson MM, Ness KK, Nolan VG, Armstrong GT, Green DM, Morris EB, et al. Prospective medical assessment of adults surviving childhood cancer: study design, cohort characteristics, and feasibility of the St. Jude Lifetime Cohort study. Pediatr Blood Cancer. 2011;56(5):825-36.

10. Hannon E, Dempster EL, Mansell G, Burrage J, Bass N, Bohlken MM, et al. DNA methylation meta-analysis reveals cellular alterations in psychosis and markers of treatment-resistant schizophrenia. Elife. 2021;10.

11. Houseman EA, Accomando WP, Koestler DC, Christensen BC, Marsit CJ, Nelson HH, et al. DNA methylation arrays as surrogate measures of cell mixture distribution. BMC Bioinformatics. 2012;13:86.

12. Wickham H. Ggplot2: Elegant graphics for data analysis. 2 ed. Cham, Switzerland: Springer International Publishing; 2016 2016/6/8.

13. Ho D, Imai K, King G, Stuart EA. MatchIt: Nonparametric Preprocessing for Parametric Causal Inference. Journal of Statistical Software. 2011;42(8):1 - 28.

14. Ritchie ME, Phipson B, Wu D, Hu Y, Law CW, Shi W, et al. limma powers differential expression analyses for RNA-sequencing and microarray studies. Nucleic Acids Res. 2015;43(7):e47.

15. Yousefi PD, Suderman M, Langdon R, Whitehurst O, Davey Smith G, Relton CL. DNA methylation-based predictors of health: applications and statistical considerations. Nat Rev Genet. 2022;23(6):369-83.

16. Christiansen C, Castillo-Fernandez JE, Domingo-Relloso A, Zhao W, El-Sayed Moustafa JS, Tsai PC, et al. Novel DNA methylation signatures of tobacco smoking with trans-ethnic effects. Clin Epigenetics. 2021;13(1):36.

17. Elliott HR, Tillin T, McArdle WL, Ho K, Duggirala A, Frayling TM, et al. Differences in smoking associated DNA methylation patterns in South Asians and Europeans. Clin Epigenetics. 2014;6(1):4.

18. Kuhn M. Building Predictive Models in R Using the caret Package. Journal of Statistical Software. 2008;28(5):1 - 26.

19. Capper D, Jones DTW, Sill M, Hovestadt V, Schrimpf D, Sturm D, et al. DNA methylation-based classification of central nervous system tumours. Nature. 2018;555(7697):469-74.

20. Krull KR, Okcu MF, Potter B, Jain N, Dreyer Z, Kamdar K, et al. Screening for neurocognitive impairment in pediatric cancer long-term survivors. J Clin Oncol. 2008;26(25):4138-43.

21. Balduzzi S, Rucker G, Schwarzer G. How to perform a meta-analysis with R: a practical tutorial. Evid Based Ment Health. 2019;22(4):153-60.

22. Yu G, Wang LG, Han Y, He QY. clusterProfiler: an R package for comparing biological themes among gene clusters. OMICS. 2012;16(5):284-7.

**MAIN TEXT REFERENCES Cont’d**

16. Van Der Plas E, Erdman L, Nieman BJ, Weksberg R, Butcher DT, O'Connor D L, et al. Characterizing neurocognitive late effects in childhood leukemia survivors using a combination of neuropsychological and cognitive neuroscience measures. Child Neuropsychol. 2018;24(8):999-1014.

17. Boulet-Craig A, Robaey P, Laniel J, Bertout L, Drouin S, Krajinovic M, et al. DIVERGT screening procedure predicts general cognitive functioning in adult long-term survivors of pediatric acute lymphoblastic leukemia: A PETALE study. Pediatr Blood Cancer. 2018;65(9):e27259.

18. Lyer NS, Balsamo LM, Bracken MB, Kadan-Lottick NS. Chemotherapy-only treatment effects on long-term neurocognitive functioning in childhood ALL survivors: a review and meta-analysis. Blood. 2015;126(3):346-53.

19. Krull KR, Brinkman TM, Li C, Armstrong GT, Ness KK, Srivastava DK, et al. Neurocognitive outcomes decades after treatment for childhood acute lymphoblastic leukemia: a report from the St Jude lifetime cohort study. J Clin Oncol. 2013;31(35):4407-15.

20. van der Plas E, Nieman BJ, Butcher DT, Hitzler JK, Weksberg R, Ito S, et al. Neurocognitive Late Effects of Chemotherapy in Survivors of Acute Lymphoblastic Leukemia: Focus on Methotrexate. J Can Acad Child Adolesc Psychiatry. 2015;24(1):25-32.

**SUPPLEMENTAL TABLES AND FIGURES**

**Table S1 Sample population characteristics**

| **Cohort/Subset (n)** | **Use in Analysis** | **% Female (n)** | **Age (median)** | **% correct classification** |
| --- | --- | --- | --- | --- |
| **NPG (140)** |  |  |  |  |
| Discovery (91) | signature discovery; model training | 29.7% (27) | 8-18 (12) | NA |
| Validation (49) | model optimization; sample classification | 55.1 % (27) | 8-19 (14) | 100% |
| **PETALE (135)** |  |  |  |  |
| Training (25) | model training | 48% (12%) | 13-29 (20) | NA |
| Testing CRT (85) | sample classification | 47% (40) | 10-38 (23) | 100% |
| Testing no CRT (25) | sample classification | 68% (17) | 14-28 (19) | 92% |
| **Controls (514)** |  |  |  |  |
| Discovery* (70) | signature discovery; model training | 40% (28) | 6-18 (13) | NA |
| Training** (50) | model training | 42% (21) | 10-28 (15) | NA |
| SickKids Testing (120) | sample classification | 52% (62) | 4-37 (14) | 88% |
| GEO Testing (274) | sample classification | 52% (143) | 18-39 (27) | 84% |
| **St. Jude LIFE (1533)** | |  |  |  |
| leukemia/lymphoma survivors (510) | sample classification | 49% (250) | 18-40 (30) | 99% |
| “other” survivors (1023) | sample classification | 47% (476) | 18-40 (29) | 77% |

*matched to NPG discovery

** matched to PETALE Training

**
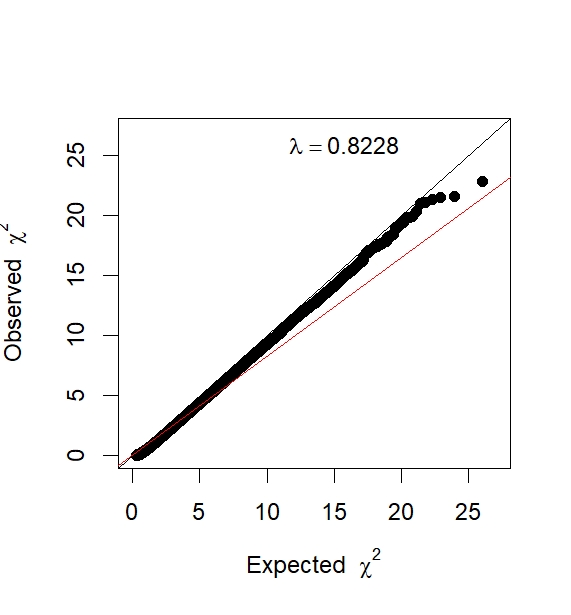
**

**Supplementary Figure S1 QQ plot of p-values generated for signature discovery using ALL survivors in the NPG discovery group and matched healthy controls.** No genomic inflation was observed (genomic inflation value = 0.8228).

**
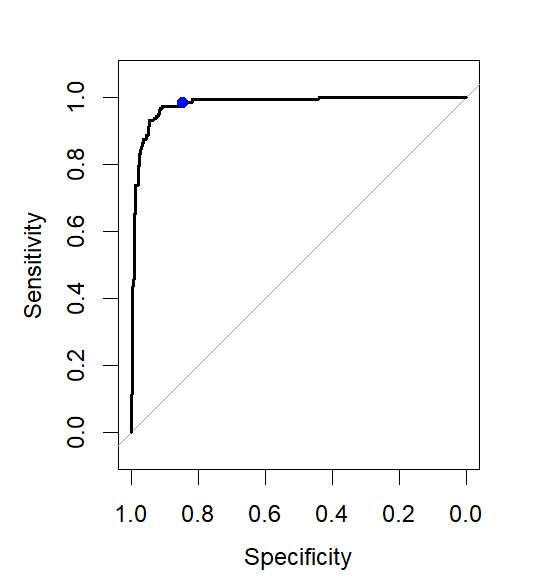
**

**Supplementary Figure S2 ROC curve of classifier when applied to all samples of ALL survivors (NPG validation, PETALE testing with and without CRT) and controls (SickKids controls, GEO controls) not used in signature discovery.** Blue point indicates a cut-off of 0.5, above which samples are classified as ALL survivors. At the threshold, sensitivity was 99% and specificity was 85%. The area under the curve was 0.98.


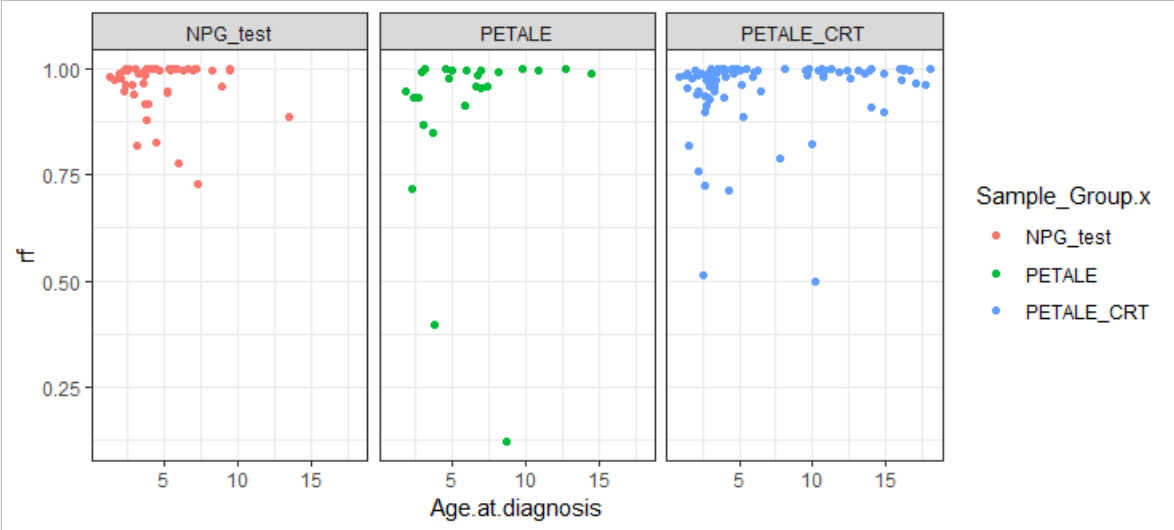


A

B

**Supplementary Figure S3 Age at samples collection (A) and age at diagnosis for ALL survivors (B) was not associated with random forest classification model scores.** Samples are grouped by cohort and the PETALE samples are further split in individuals treated with or without cranial radiation therapy (CRT).


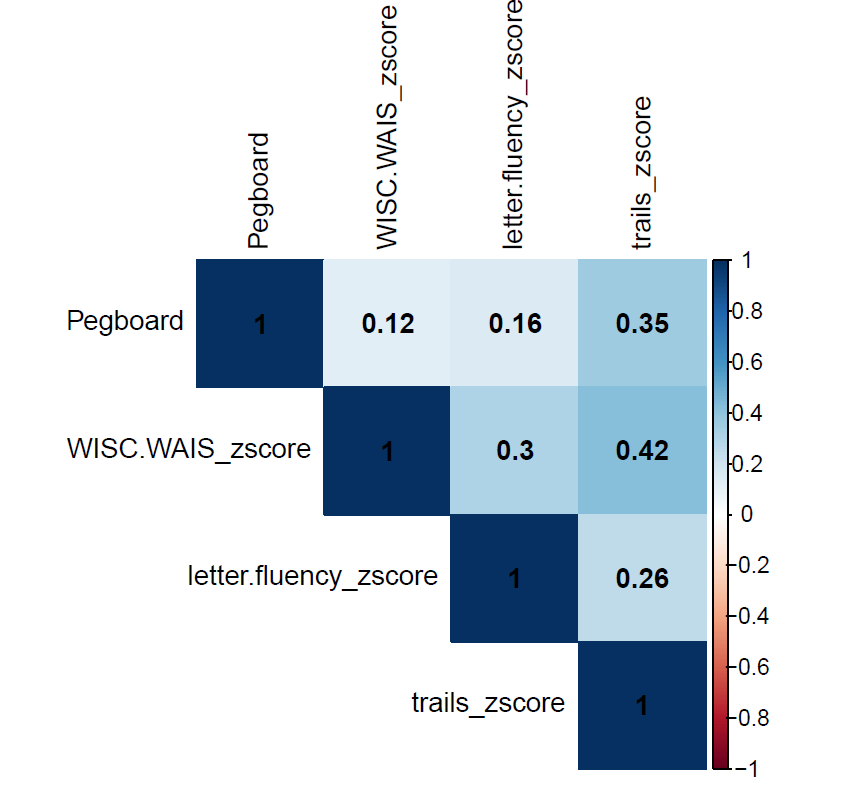

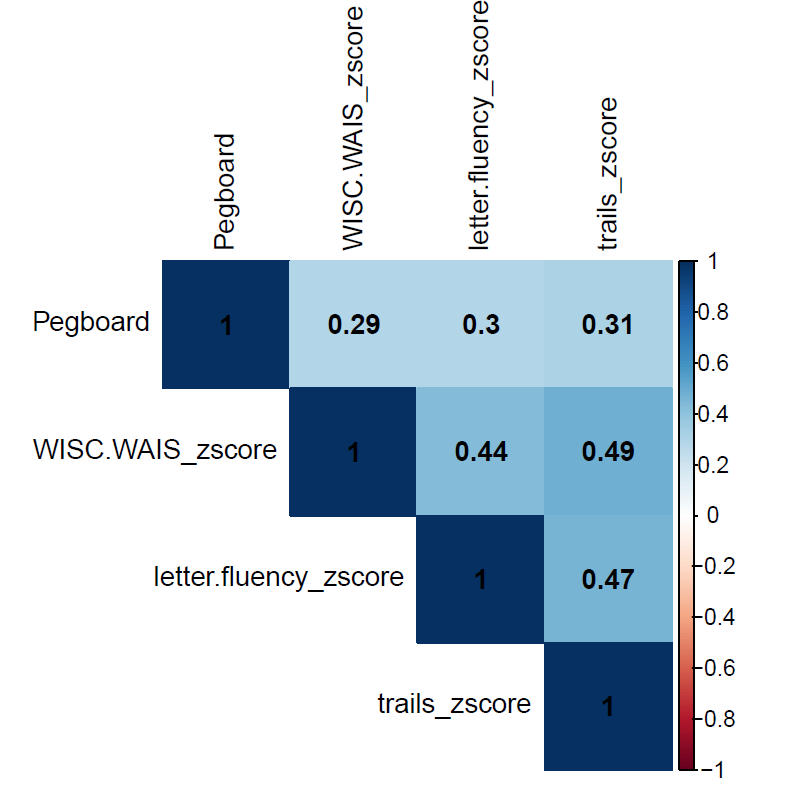


NPG

PETALE

A

B

**Supplementary Figure S4 Correlations of DIVERGT test z-scores within NPG (A) and PETALE (B).**


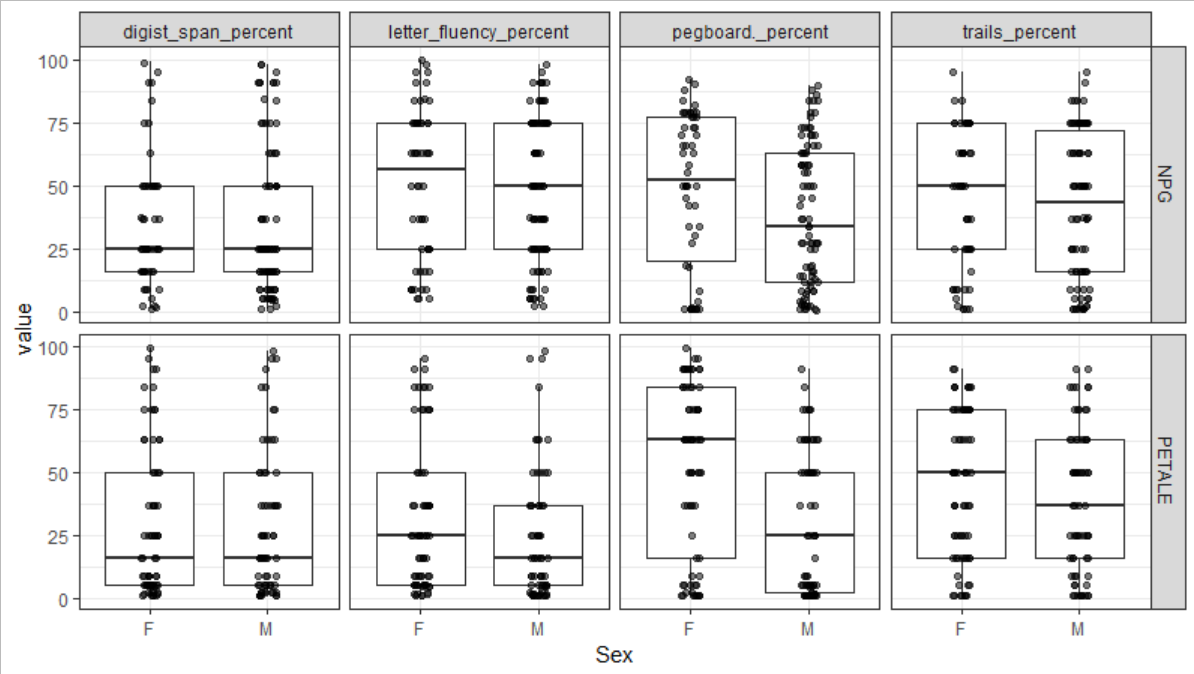


*

**Supplementary Figure S5 Percentiles for DIVERGT test in males and females in each cohort.** Grooved Pegboard score was significantly higher in females in the PETALE cohort (p<0.05) but not NPG.
